# Demonstrating specificity of bioactive peptide nucleic acids (PNAs) targeting microRNAs for practical laboratory classes of applied biochemistry and pharmacology

**DOI:** 10.1371/journal.pone.0221923

**Published:** 2019-09-11

**Authors:** Jessica Gasparello, Chiara Papi, Matteo Zurlo, Roberto Corradini, Roberto Gambari, Alessia Finotti

**Affiliations:** 1 Department of Life Sciences and Biotechnology, University of Ferrara, Ferrara, Italy; 2 Department of Chemistry, Life Sciences and Environmental Sustainability, University of Parma, Parma, Italy; 3 Interuniversity Consortium for Biotechnology (CIB), Trieste, Italy; Helsingin Yliopisto, FINLAND

## Abstract

Practical laboratory classes teaching molecular pharmacology approaches employed in the development of therapeutic strategies are of great interest for students of courses in Biotechnology, Applied Biology, Pharmaceutic and Technology Chemistry, Translational Oncology. Unfortunately, in most cases the technology to be transferred to learning students is complex and requires multi-step approaches. In this respect, simple and straightforward experimental protocols might be of great interest. This study was aimed at presenting a laboratory exercise focusing (a) on a very challenging therapeutic strategy, i.e. microRNA therapeutics, and (b) on the employment of biomolecules of great interest in applied biology and pharmacology, i.e. peptide nucleic acids (PNAs). The aims of the practical laboratory were to determine: (a) the possible PNA-mediated arrest in RT-qPCR, to be eventually used to demonstrate PNA targeting of selected miRNAs; (b) the possible lack of activity on mutated PNA sequences; (c) the effects (if any) on the amplification of other unrelated miRNA sequences. The results which can be obtained support the following conclusions: PNA-mediated arrest in RT-qPCR can be analyzed in a easy way; mutated PNA sequences are completely inactive; the effects of the employed PNAs are specific and no inhibitory effect occurs on other unrelated miRNA sequences. This activity is simple (cell culture, RNA extraction, RT-qPCR are all well-established technologies), fast (starting from isolated and characterized RNA, few hours are just necessary), highly reproducible (therefore easily employed by even untrained students). On the other hand, these laboratory lessons require some facilities, the most critical being the availability of instruments for PCR. While this might be a problem in the case these instruments are not available, we would like to underline that determination of the presence or of a lack of amplified product can be also obtained using standard analytical approaches based on agarose gel electrophoresis.

## Introduction

Large number of students engaged in scientific disciplines are expected to very interested in authentic laboratories experiences in molecular biology classrooms [1,2]. Accordingly, practical laboratory classrooms based on teaching molecular pharmacology approaches employed in the development of therapeutic strategies are of great interest for students of courses in Biotechnology, Applied Biology, Pharmaceutic and Technology Chemistry, Translational Oncology. Unfortunately, despite several experiences are important, they are challenging as, in most of the cases, the technology to be transferred to learning students is complex and requires multi-step approaches [3]. Furthermore, several technologies require complex instrumentation(s) and costly reagents and supplies [3]. Finally, several techniques are difficult to be followed in in real lab (wet-lab) setting in the case of large class size resulting in student crowding [4]. Based on these considerations virtual laboratories have been proposed, which facilitate learning of technologies requiring complex instruments, costly reagents and materials, highly trained personnel [2, 5–8].

However, we should underline that the student’s expectation might require also the organization of wet-labs for acquiring complex skills and the ability to discuss challenging biomedical approaches [9,10]. In this respect, simple and straightforward experimental protocols might be useful and of great interest, especially in the era of personalized medicine and molecular targeting.

This study is aimed at presenting a laboratory exercise focusing (a) on a very challenging therapeutic strategy, i.e. microRNA therapeutics [11–14], and (b) on the employment of biomolecules of great interest in applied biology and pharmacology, i.e. Peptide Nucleic Acids (PNAs) [15–17].

MicroRNAs (miRNAs) are a family of evolutionary conserved small (19 to 25 nucleotides in length) noncoding RNAs playing important roles in the post-transcriptional control of gene expression, operated at the level of mRNA translation and based on the miRNA-dependent recognition of 3’UTR, CDS and 5’UTR mRNA sequences [18–22]. Excellent reviews on miRNA biology are available and might be considered in the teaching materials available to the students [23,24]. A second point is that microRNAs are novel and very important targets for therapeutic strategies [13,14, 25–28]; the anti-miRNA and miRNA replacement approaches to modify miRNA-regulated gene expression are summarized in Figure A in S1 File). In this study we focused on the teaching of methods for characterize the specificity of biomolecules to be employed in anti-miRNA strategies [29–44].

The considered biomolecule are based on Peptide Nucleic Acids, DNA analogues described for the first time by Nielsen et al. [45], in which the sugar-phosphate backbone has been replaced by N-(2-aminoethyl)glycine units [15–17,46] as depicted in Figure B in S1 File. PNAs have been demonstrated to be very efficient tools for pharmacologically-mediated alteration of gene expression, both *in vitro* and *in vivo* [47–49], in consideration of the possibility to be used as antisense molecules targeting mRNAs, triple-helix forming molecules targeting eukaryotic gene promoters, artificial promoters, decoy molecules targeting transcription factors [15–17,46–49]. Relevant in the context of the proposed practical laboratory exercise, PNAs have been demonstrated to be able of altering miRNA functions, both *in vitro* and *in vivo* [50–58]. This has been recently reviewed by Manicardi et al. [57] and more information is depicted in the Figure C in S1 File.

The protocol here presented considers two PNAs, a PNA targeting miR-221-3p (R8-PNA-a221) and causing activation of apoptosis of treated cancer cells through inhibition of miR-221-3p functions, and a PNA targeting miR-145-5p (R8-PNA-a145), able to induce in increase of the expression of CFTR through inhibition of the CFTR regulator: miR-145-5p.

We have recently found that a PNA targeting miR-221-3p (R8-PNA-a221) [53], bearing an oligoarginine peptide (R_8_) enabling efficient uptake by glioma cells [52,55,59], was able to strongly inhibit miR-221-3p in U251, U373 and T98G glioma cells. This inhibition of miR-221-3p activity was associated with increased expression of the miR-221 target p27^Kip1^, analyzed by RT-qPCR and by Western blotting [52,60] (see Figure D in S1 File). As far as targeting miR-145-5p, we have described a PNA against miR-145-5p which inhibits the activity of the target miRNA and enhances the expression of the miR-145-5p regulated Cystic Fibrosis Transmembrane Conductance Regulator (CFTR) in Calu-3 Cells [61,62] (see Figure E in S1 File).

## Materials and methods

### Materials

#### Peptide nucleic acids (PNAs)

PNAs can be purchased from several companies, including Panagene Inc. (www.panagene.com; Yuseong-gu, Daejeon, South Korea). Alternatively, PNAs against miRNAs can be synthesized following the procedures described in Manicardi et al. [57] and Fabbri et al. [61]. The data here presented are based on PNAs described in the paper by Brognara et al. [52]. The sequences of PNA-a221 and PNA-a145 are reported in Table 1; PNAs with a mutated sequence were used as negative controls.

**Table 1:** Sequences of PNAs. Mutated bases are underlined.

PNA name	Sequence
**R8-PNA-a221**	H-RRRRRRRR-AAACCCAGCAGACAATGT-Gly-NH_2_
**R8-PNA-a221-MUT**	H-RRRRRRRR-AA**T**CCCA**C**CAGA**G**AA**A**GT-Gly-NH_2_
**R8-PNA-a145**	H-RRRRRRRR-AGGGATTCCTGGGAAAAC-Gly-NH_2_
**R8-PNA-a145-MUT**	H-RRRRRRRR-AG**A**GAT**G**CCT**T**GGA**G**AAC-Gly-NH_2_

#### Cell lines

1. U251 human glioma cell line (Sigma-Aldrich, St.Louis, Missouri, USA; cat.09063001)

2. Calu-3 human airway epithelial Calu-3 cell line (American Type Culture Collection: ATCC HTB-55)

#### Cell culture

1. RPMI-1640 medium (Sigma-Aldrich, St.Louis, Missouri, USA)

2. D-MEM medium (Gibco, Thermo Fisher Scientific, Walthman, Massachusetts, USA)

3. 100 U/mL penicillin and 100 μg/mL streptomycin (Sigma-Aldrich, St.Louis, Missouri, USA)

4. Fetal bovine serum (FBS, Biowest, Nauillè, France)

5. Non-Essential Amino Acids Solution 100X (NEAA, Gibco, Thermo Fisher Scientific, Walthman, Massachusetts, USA)

6. To determine cell growth a Z2 Coulter Counter (Coulter Electronics, Hialeah, Florida, USA)

#### RNA extraction

1. Trypsin-EDTA (Sigma-Aldrich, St.Loius Missouri, USA)

2. FBS (FBS, Biowest, Nauillè, France)

3. DPBS (Gibco, Thermo Fisher Scientific, Walthman, Massachusetts, USA)

4. Tri-Reagent (Sigma-Aldrich, St.Loius Missouri, USA) was employed for cell lysis

5. Extracted RNA was quantified using SmartSpec Plus Spectrophotometer (Bio-Rad, Hercules, CA, USA)

6. The quality of the RNA was determined by spectrophotometric analysis and by agarose gel electrophoresis

#### MicroRNA reverse transcription reaction

1. Specific stem loop primers for miRNA Reverse Transcription (RT) have been purchased from Applied Biosystems (Thermo Fisher Scientific, Walthman, Massachusetts, USA). The ID of employed assays (including primers and probes for RT-qPCR and RT-ddPCR reaction) have been reported in Table 2.

**Table 2:** Assays employed for miRNA quantification by RT-qPCR and RT-ddPCR.

miRNA	Assay number	MicroRNA sequence
*hsa-miR-221-3p*	000524	5’-AGCUACAUUGUCUGCUGGGUUUC-3’
*hsa-miR-222-3p*	002276	5’-AGCUACAUCUGGCUACUGGGU-3’
*hsa-miR-210-3p*	000512	5’-CUGUGCGUGUGACAGCGGCUGA-3’
*hsa-miR-145-5p*	002278	5’-GUCCAGUUUUCCCAGGAAUCCCU-3’
*hsa-miR-155-5p*	002623	5’-UUAAUGCUAAUCGUGAUAGGGGUU-3’
*hsa-let-7c-5p*	000379	5’-UGAGGUAGUAGGUUGUAUGGUU-3’

2. Reverse transcriptase (RT) reactions were performed using the TaqMan MicroRNA Reverse Transcription Kit (Thermo Fisher Scientific, Walthman, Massachusetts, USA).

3. MicroRNA reverse transcription reaction was performed using GeneAmp PCR System 9700 (Thermo Fisher Scientific, Walthman, Massachusetts, USA)

#### Real-time quantitative PCR of microRNA

1. Primers and probes for miRNA amplification have been obtained from Applied Biosystems (Thermo Fisher Scientific, Walthman, Massachusetts, USA). The ID of employed assays has been reported in Table 2.

2. All RT-qPCR reactions were conducted using TaqMan Universal PCR Master Mix, no AmpErase UNG (Thermo Fisher Scientific, Walthman, Massachusetts, USA)

3. Real-time PCR was performed using the CFX96 Touch Real-time PCR Detection System (Bio-Rad, Hercules, CA, USA) and data collection and analysis was performed using CFX Manager Software version 3.1 (Bio-Rad, Hercules, CA, USA).

#### Droplet Digital PCR (ddPCR) analysis of microRNA

1. Primers and probes for miRNA amplification have been obtained from Applied Biosystems (Thermo Fisher Scientific, Walthman, Massachusetts, USA). The ID of employed assays has been reported in Table 2.

2. DNA polymerase and the necessary reagents for miRNA amplification are contained in ddPCR Supermix for Probes (No dUTP) (Bio-Rad, Hercules, CA, USA)

3. Water in oil emulsion was automated created using Automated Droplet Generator (AutoDG, Bio-Rad, Hercules, CA, USA), DG32 Automated Droplet Generator Cartridges (Bio-Rad, Hercules, CA, USA) and Automated Droplet Generation Oil for Probes (Bio

Rad, Hercules, CA, USA)

4. MicroRNA amplification was performed using GeneAmp PCR System 9700 (Thermo Fisher Scientific, Walthman, Massachusetts, USA)

5. Generated droplets were analyzed using QX200 Droplet Reader (Bio-Rad, Hercules, CA, USA) and data analysis was performed using QuantaSoft version 1.7.4 (Bio-Rad, Hercules, CA, USA)

### Methods

#### Human cell lines and culture conditions

U251 and Calu-3 cells were cultured in humidified atmosphere of 5% CO2/air. U251 glioma cell line were maintained in culture medium composed by RPMI-1640 (Sigma-Aldrich) and 10% FBS (Biowest) supplemented with 100 units/mL penicillin and 100 g/mL streptomycin, while Calu-3 were cultured in D-MEM medium (Gibco) supplemented with 10% fetal bovine serum, 100 units/mL penicillin and 100 g/mL streptomycin and 1% NEAA (100x) (Non-Essential Amino Acids Solution; Gibco).

#### Total RNA extraction

Cells were trypsinized and collected by centrifugation at 1500 RPM for 10 min at 4°C, washed with DPBS and lysed with Tri-Reagent (Sigma-Aldrich), according to manufacturer’s instructions. The isolated RNA was washed once, with cold 75% ethanol, dried and dissolved in nuclease free water (Sigma-Aldrich) before use. The obtained RNA was stored at -80°C until the use. The quality of the RNA was determined by spectrophotometric analysis and by agarose gel electrophoresis. The ratio 260/280 nm was used for determining the overall quality. The electrophoresis on 0,8% agarose in TAE (Tris-acetate-EDTA) buffer was employed for quality checking.

#### MicroRNA reverse transcription

Obtained total RNA was quantified using SmartSpec Plus Spectrophotometer (Bio-Rad) and 300 ng of total RNA were reverse transcribed using TaqMan MicroRNA Reverse Transcription Kit (Thermo Fisher Scientific) and specific stem loop primers (Thermo Fisher Scientific) following manufacturer instructions. Obtained miRNA-specific cDNA was stored at -80°C until PCR analysis.

#### Real-time quantitative PCR of microRNA

Three μL of obtained cDNA were amplified in 25 μL (final volume) of RT-qPCR reaction mix, containing 2X TaqMan Universal PCR Master Mix, no AmpErase UNG (Thermo Fisher Scientific) and 20X TaqMan MicroRNA Assay (Thermo Fisher Scientific) indicated in Table 2. Incremental concentrations (from 25 nM to 200 nM) of anti-miR PNAs were added to the RT-qPCR reaction mix of PNA treated mix, while no PNA was added to the control samples. Sequences of the employed PNAs are reported in Table 1. All RT-qPCR reactions, including no-template controls (NTC) and RT-minus controls, were run in duplicate, using the CFX96 Touch Real Time PCR Detection System (Bio-rad). Data analysis and graphic elaborations were performed using CFX Manager Software version 3.1 (Bio-Rad).

#### Droplet Digital PCR analysis of microRNA

The ability of PNA to arrest miRNA amplification reaction was also tested using ddPCR; at this purpose 1 μL of 1:50 diluted cDNA obtained from U251 cell line was added to ddPCR reaction mix containing 2X ddPCR Supermix for Probes (no dUTP) (Bio-Rad) and 20X TaqMan MicroRNA Assay (Thermo Fisher Scientific). In this case three different concentration anti-miR PNAs were employed: 25, 50 and 100 nM, while no PNA was added to control samples. 20 μL of ddPCR reaction mix were mixed with Automated Droplet Generation Oil for Probes (Bio-Rad) and 40 μL of droplets emulsion was automatically generated using Automated Droplet Generator (AutoDG) (Bio-Rad). The emulsion was amplified using GeneAmp PCR System 9700 (Thermo Fisher Scientific) using the following thermal cycler condition 95°C for 10 min, 40 cycles of 95°C for 15 s and 60°C for 1 min and a final step of 98°C for 10 min. Genereted droplets were read using the QX200 Droplet Reader, and data analysis was performed with QuantaSoft version 1.7.4 (Bio-Rad).

#### Statistics

Results are expressed as mean ± standard error of the mean (SEM). Comparisons between groups were made by using paired Student's *t* test and a one-way analysis of variance (ANOVA). Statistical significance was defined with *p*<0.01.

## Results

### Cell culture and RNA extraction

The objective of these procedures is to obtain the RNA samples to be employed by the students during the practical laboratory. Depending on the time and the program, it can be also included as a part of the lesson(s). U251 human glioma [63,64] and Calu-3 human airway epithelial [65,66] cell lines can be cultured in humidified atmosphere of 5% CO_2_/air in D-MEM medium (Gibco) supplemented with 10% fetal bovine serum (Biowest), 100 units/mL penicillin and 100 μg/mL streptomycin and 1% NEAA (100X) (Non-Essential Amino Acids Solution, Gibco). For RNA extraction cultured cells are trypsinized and collected by centrifugation at 1500 rpm for 10 minutes at 4°C, washed with cold DPBS (Gibco), lysed with Tri-Reagent (Sigma-Aldrich), according to manufacturer’s instructions. The isolated RNA is washed once with cold 75% ethanol, dried and dissolved in nuclease-free water (Sigma-Aldrich). The quality of the RNA was determined by spectrophotometric analysis and by agarose gel electrophoresis. The ratio 260/280 nm was used for determining the overall quality (representative examples are shown in Figure F in S1 File). Agarose gel electrophoresis was employed for quality checking (representative examples are shown in Figure F in S1 File).

### Functions of miR-221-3p and miR-145-5p in U251 and Calu-3 cells

The reason for selecting miR-221-3p and miR-145-5p as miRNA PNA targets is related to previously published studies demonstrating that PNA-mediated inhibition of the activity of these two miRNAs is associated with clinically relevant effects. Brognara et al. [53] reported that a PNA against miR-221-3p is able to induce apoptosis of the treated glioma cell line. More recently Brognara et al. [54] demonstrated that two PNAs, one against miR-221, the other against miR-222-3p were able to induced higher levels of apoptosis when administered to the glioma cells in combination. These results are relevant in the development of PNA-based miRNA targeting in experimental oncology. The key experiments of these studies can be presented as a background to the class using the Figure D in S1 File. As far as miR-145-5p, Fabbri et al. [61] proposed the use of an anti-miR PNA for targeting miR-145-5p, a microRNA reported to suppress the expression of the Cystic Fibrosis Transmembrane conductance Regulator (CFTR) gene. Sequence dependent targeting of miR-145-5p was demonstrated in Calu-3 cells, allowing to enhance expression of the miR-145-5p regulated CFTR gene, analyzed at mRNA (RT-qPCR) and protein (western blotting) level. These results are relevant in the development of PNA-based miRNA targeting for cystic fibrosis [62,67,68]. The key experiments of these studies can be presented as a background to the class using the Figure E in S1 File.

### Outline of the practical laboratory program

The outline of the main practical laboratory, starting from the isolated RNA described in section 3.1, will answer to the following questions:

- Are PNAs (PNA-a221 and PNA-a145) able to arrest RT-qPCR designed for the amplification of the PNA-target miRNA sequences (miR-221-3p for PNA-a221 and miR-145-5p for PNA-a145)?
- Are mutated PNAs (PNA-a221-MUT and PNA-a145-MUT) active?
- Are unrelated miRNA sequences amplified in the presence of PNA-a221 and PNA-a145, supporting selectivity of PNA-mediated effects?

### Effects of PNA-a221 on RT-qPCR amplification of miR-221-3p sequences

**Fig 1:**
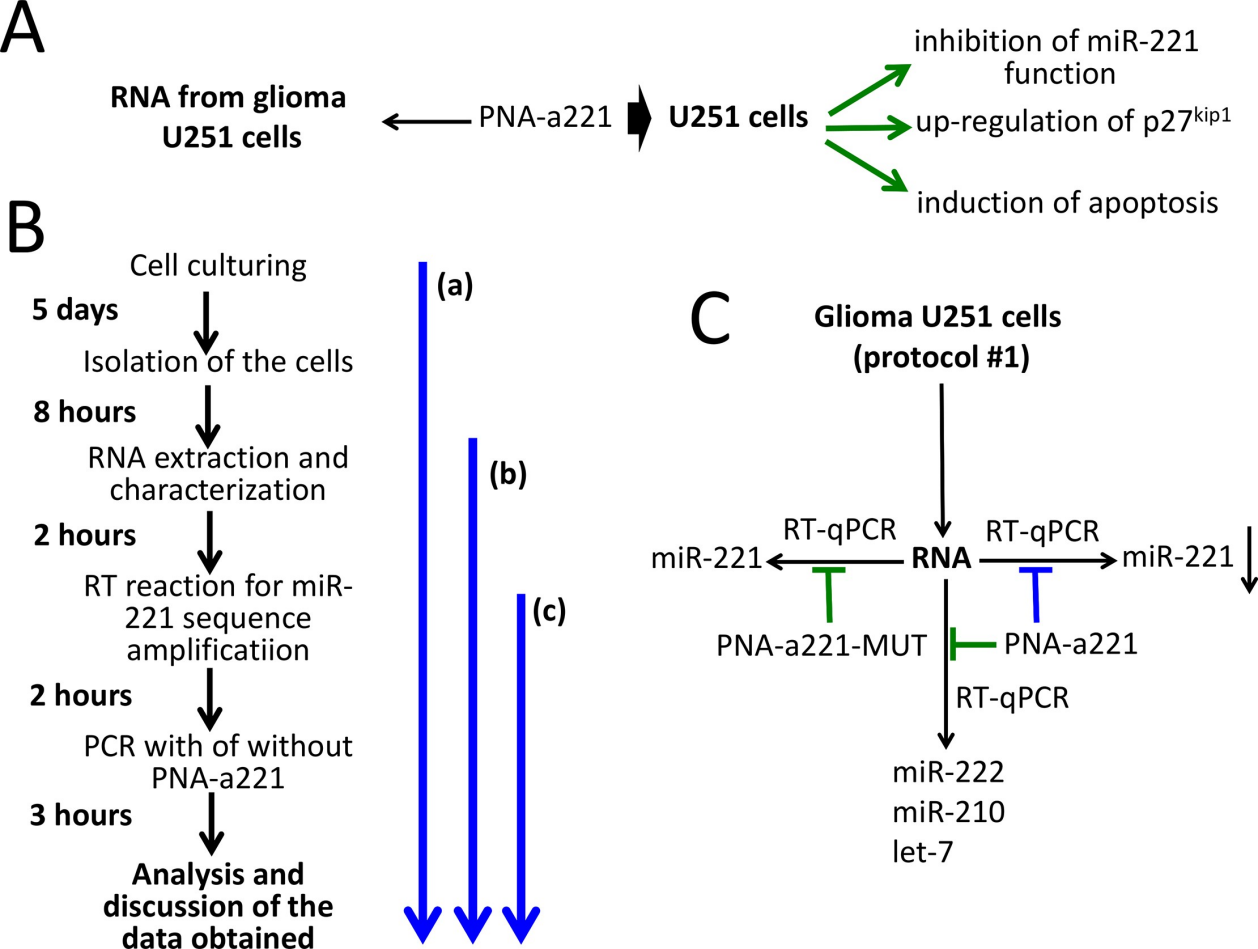
Biological effects of a PNA targeting miR-221-3p and outline of the practical laboratory program. A. Scheme of the background available in the literature on the biological effects of the R8-PNA-a221 on human glioma cell lines (Fabbri et al, 2017). More detailed information is shown in Figure A in S1 File. B. Timing of the laboratory practice, depending on the starting activity (identified by the blue arrows). The key activity is shown as the segment (c). Alternatively, U251 cell culture and RNA extraction/characterization (a) or only RNA extraction (b) might be considered. C. Scheme of the laboratory practice finalized to verify the specificity of the biological activity of the PNA-a221. The extracted U251 RNA is used for RT-qPCR in the presence of the PNA-a221 and the PNA-a221-MUT. The amplified miRNAs are indicated. Expected results (blue: inhibition; green: no inhibition) when PNA-a221 and PNA-a221-MUT are employed and miR-221, miR-222, miR-210 and let-7 sequences amplified by RT-qPCR. Specificity can be demonstrated if inhibition of the RT-qPCR product is obtained amplifying miR-221-3p in the presence of PNA-a221.

Fig 1 shows the outline of the experiments based on the use of PNA-a221. In Fig 1A the biological effects of the R8-PNA-a221 on glioma cell lines are summarized (see also Figure D in S1 File). Fig 1B shows the timing of the proposed practical laboratory activity. The key activity is shown as the segment **(c)** of Fig 1B and starts from the RNA preparation, is based on the performing of the PCR following RT in the absence or in the presence of PNA-a221 (as further depicted in Fig 1C). Alternatively, U251 cell culture and RNA extraction/characterization—segment **(a)** of Fig 1B—or only RNA extraction—segment **(b)** of Fig 1B—might be considered for inclusion in the practical teaching protocol, depending on the available time.

**Fig 2:**
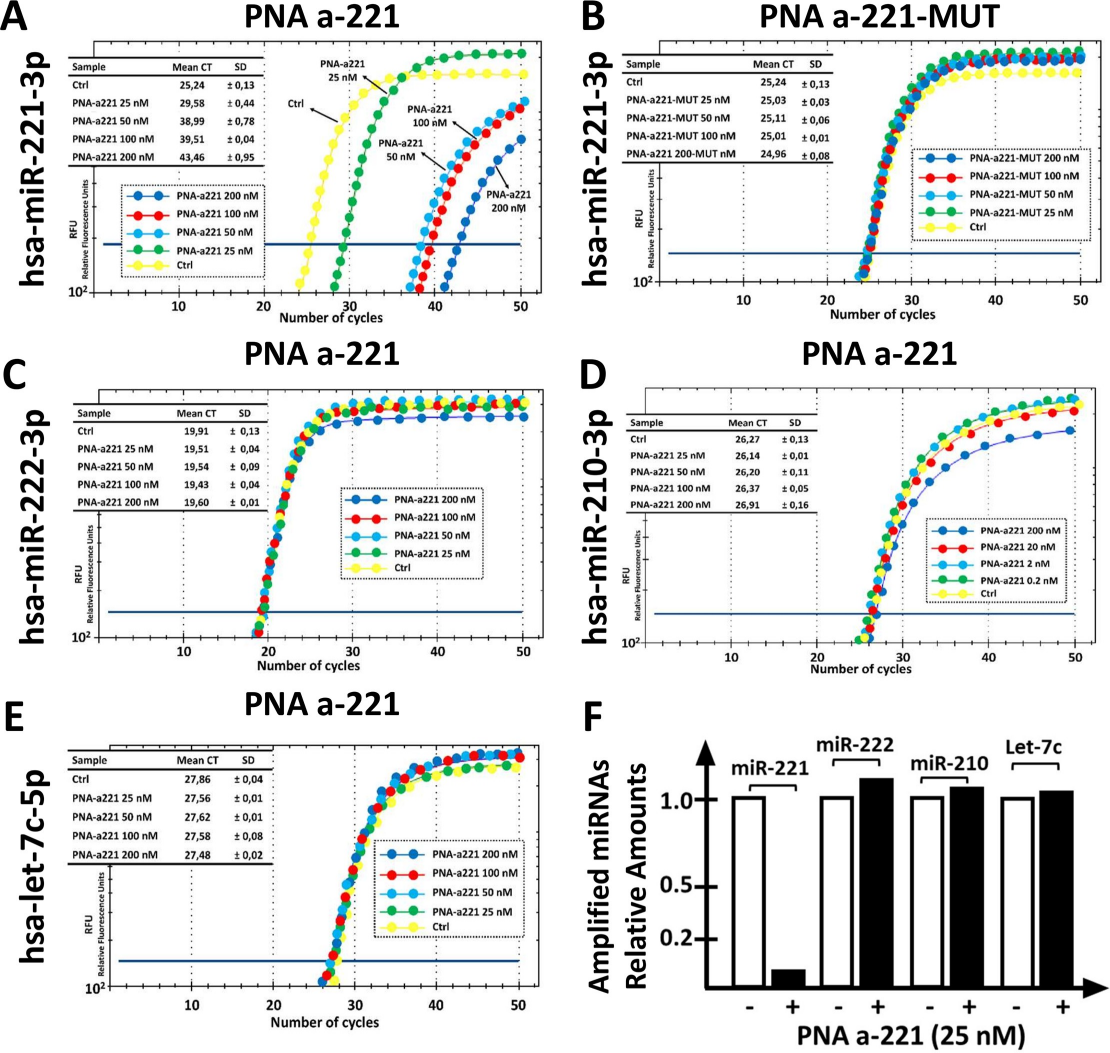
Effects of the PNA-a221 on the RT-PCR amplification of miRNA sequences. A,B. Effects of increasing amounts of PNA-a221 (A) and mutated PNA-a221-MUT (B) on the amplification of miR-221-3p sequences. C-E. Effects of increasing amounts of PNA-a221 on the amplification of miR-222-3p (C), miR-210-3p (D) and let-7c-5p (E) sequences. F. Summary of the effects of 25 nM PNA-a221 on amplification of the indicated miRNA sequences. The comparison of the effects of 50, 100 and 200 nM PNA-a221 are presented in Figure E in S1 File.

The first set of key results that can be obtained during this practical exercise are shown in Fig 2, panel A and B. As clearly evident, increasing concentrations of the R8-PNA-a221 have dramatic inhibitory effects on the RT-qPCR amplification of miR-221-3p sequences. It can be easily concluded that 25 nM PNA is sufficient to cause a 75% inhibition of PCR amplification (Fig 2A). On the contrary, the mutated R8-PNA-a221 (for the sequence of the R8-PNA-a221 and R8-PNA-a221-MUT see Table 1) is completely inactive, even when added at 200 nM (Fig 2A). This first set of results was obtained with high levels of reproducibility, obtaining highly significant values when the data concerning treatments with R8-PNA-a221 and R8-PNA-a221-MUT are compared (p <0.0000123 in five independent determinations. The second part of this laboratory exercise is considered in Fig 2, panels C-E. The results obtained demonstrate that the treatment with the R8-PNA-a221 has no effects of the RT-qPCR amplification of miR-222-3p, miR-210-3p and miR let-7c-5p sequences. Altogether, these data are consistent with the hypothesis that the effects of R8-PNA-a221 are highly specific. Of particular interest are the data demonstrating that the R8-PNA-a221 has no effect on the RT-qPCR amplification of miR-222-3p, which shares with miR-221-3p extensive sequence homologies and similar biological effects. The summary of the effects of 25 nM R8-PNA-a221 is shown in Fig 2F, all the quantitative data in Figure G in S1 File.

### Effects of PNA-a145 on RT-qPCR amplification of miR-145-5p sequences

**Fig 3:**
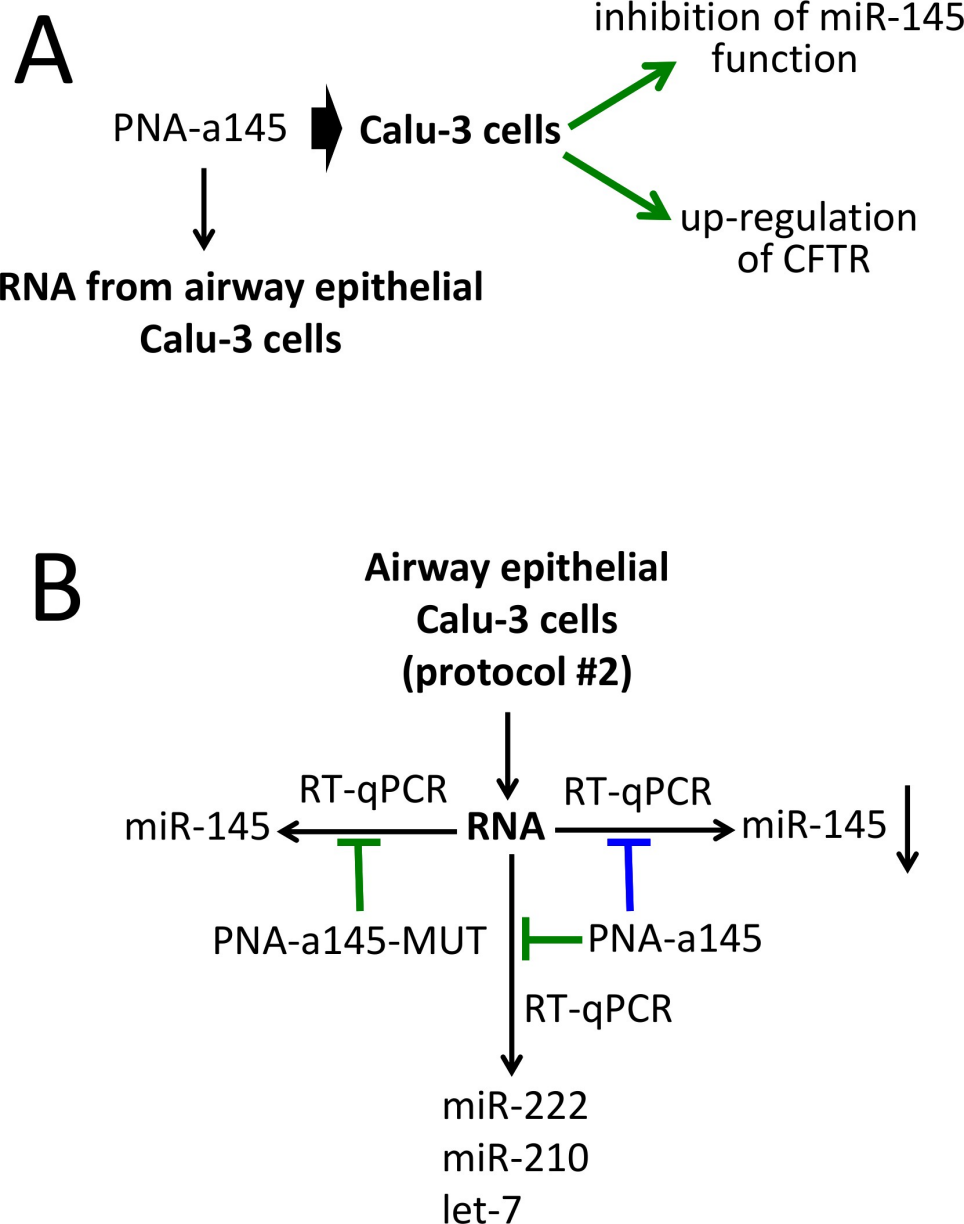
Biological effects of a PNA targeting miR-145-5p and outline of the practical laboratory program. A. Scheme of the background available in the literature on the biological effects of the R8-PNA-a145 on the Calu-3 cell line (Fabbri et al., 2017). More detailed information is shown in Figure D in S1 File. B. Scheme of the laboratory practice finalized to verify the specificity of the biological activity of the PNA-a145. The extracted Calu-3 RNA is used for RT-qPCR in the presence of the PNA-a145 and the PNA-a145-MUT (green: no inhibition; blue: inhibition). The amplified miRNAs (miR-145-5p, let-7c-5p and miR-155-5p) are indicated. Specificity can be demonstrated if inhibition of the RT-qPCR product is obtained amplifying miR-145-5p in the presence of PNA-a145.

Fig 3 shows the outline of the experiments based on the use of PNA-a145. In Fig 3A the biological effects of the R8-PNA-a145 on Calu-3 cells (see also Figure E in S1 File) are summarized. The timing of the proposed practical laboratory activity is similar to that reported in Fig 1B for the R8-PNA-a221. The key activity is shown in Fig 3B.

**Fig 4:**
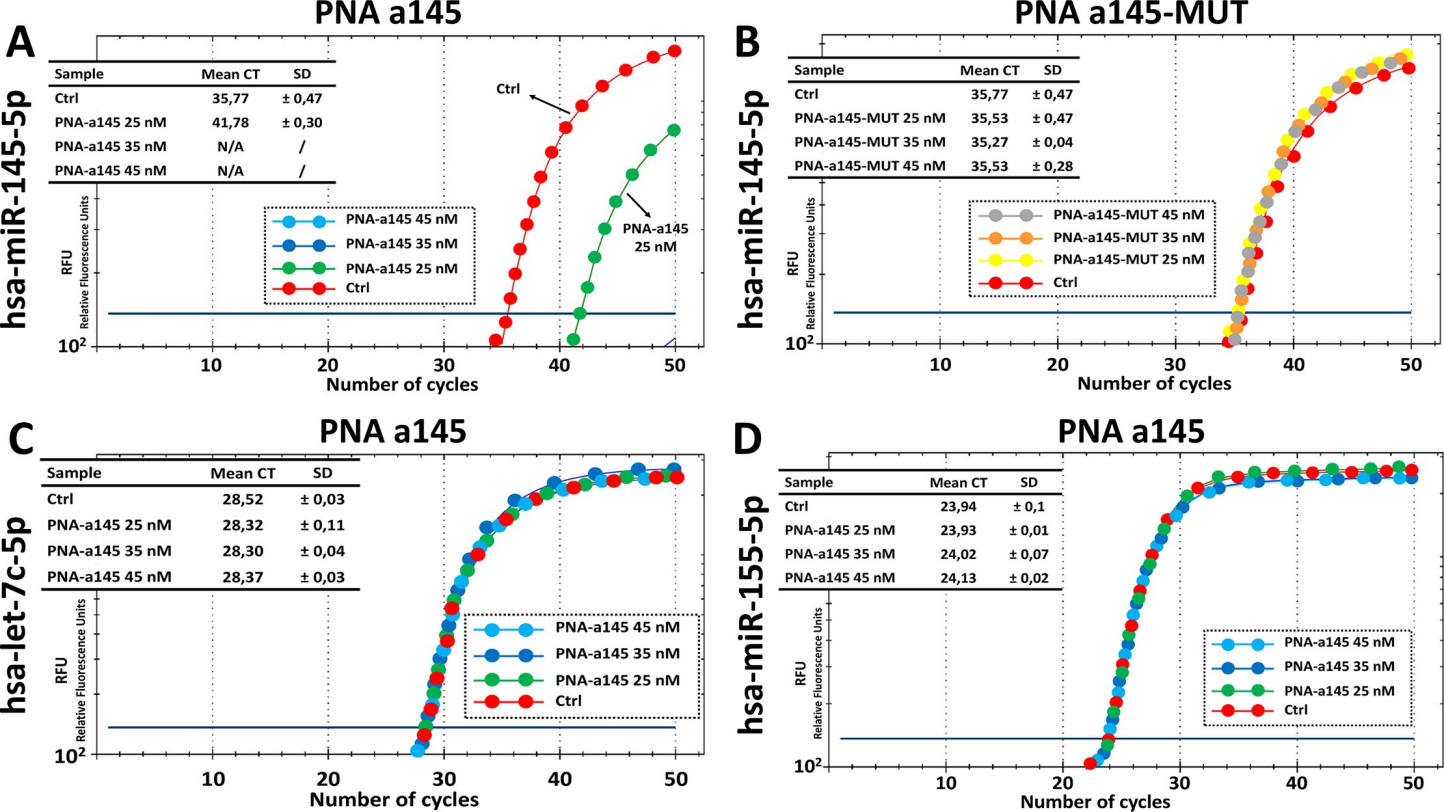
Effects of the PNA-a145 on the RT-PCR amplification of miRNA sequences. A,B. Effects of 25 nM PNA-a145 (A) and mutated PNA-a145-MUT (B) on the amplification of miR-145-5p sequences. C,D. Effects of 25 nM PNA-a145 on the amplification of miR-155-5p (C) and let-7c-5p (D) sequences. A summary of increasing concentrations of PNA-a145 are presented in Figure F in S1 File.

The first key results that can be obtained during this practical exercise are shown in Fig 4, panel A and B. As clearly evident, the R8-PNA-a145 has dramatic inhibitory effects on the RT-qPCR amplification of miR-145-5p sequences. It can be easily concluded that 25 nM PNA is sufficient to cause a 90% inhibition of PCR amplification (Fig 4A). On the contrary, the mutated R8-PNA-a145 (for the sequence of the R8-PNA-a145 and R8-PNA-a145-MUT see Table 1) is completely inactive (Fig 4B). The second part of this laboratory exercise is considered in Fig 4, panels C and D. The results obtained demonstrate that the treatment with the R8-PNA-a145 has no effects of the RT-qPCR amplification of miR-155-5p and let-7c-5p sequences. Altogether, these data are consistent with the hypothesis that the effects of R8-PNA-a145 are highly specific. The summary of the effects of different concentrations of R8-PNA-a145 is shown in Figure H in S1 File).

### Employment of other RT-PCR systems: Droplet digital PCR

**Fig 5:**
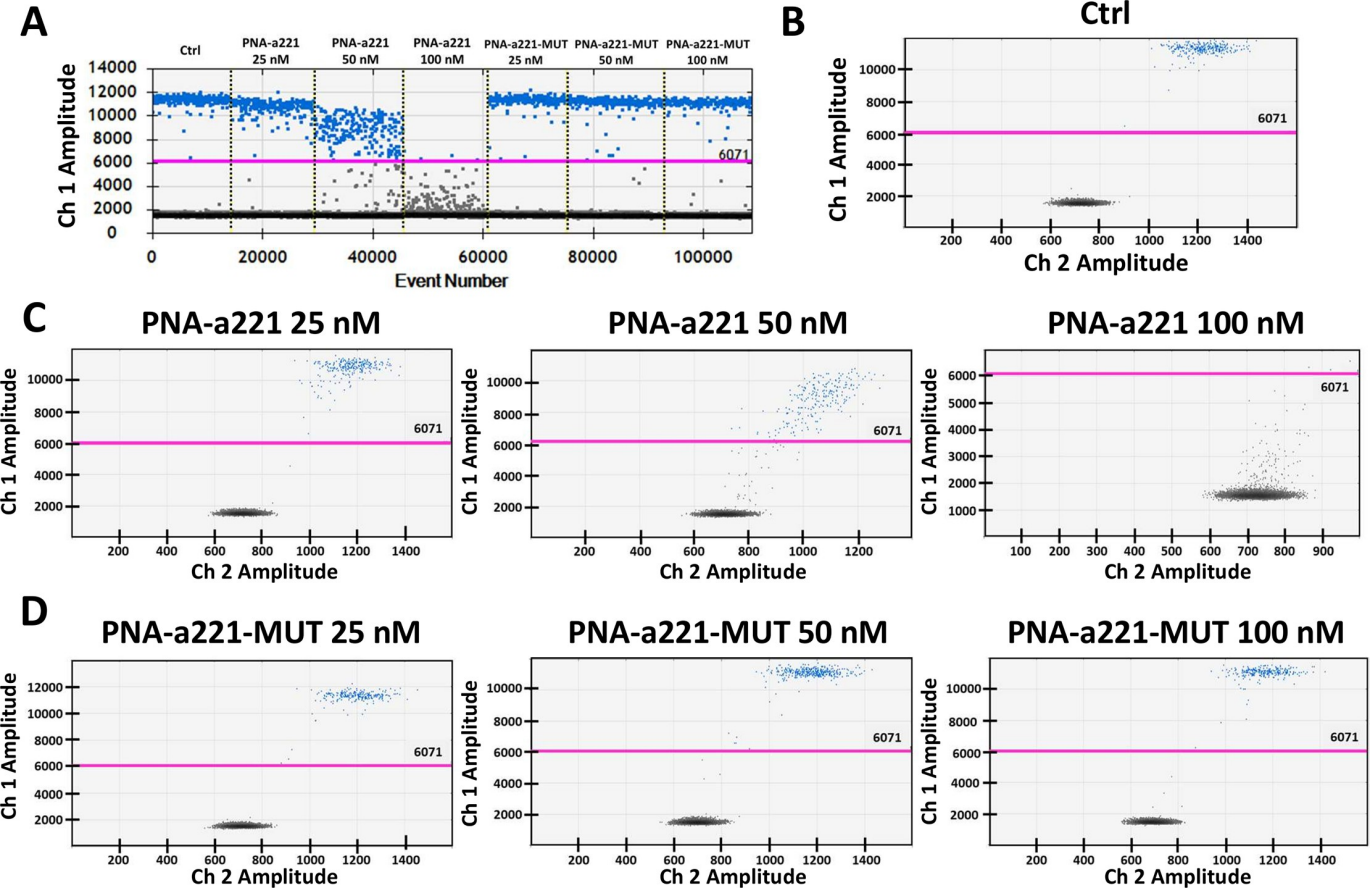
Effects of R8-PNA-a221 on miR-221-3p sequence detection by RT-ddPCR. A. 1D RT-ddPCR plot obtained after the addition of incremental concentration of R8-PNA-a221 or R8-PNA-a221-MUT to the reaction mix. B. miR-221-3p content in 1:50 diluted cDNA obtained from U251 cells: 2D plots. C. miR-221-3p content detected after the addition of incremental R8-PNA-a221concentrations. D. miR-221-3p content detected after the addition of incremental R8-PNA-a221-MUT concentrations.

**Fig 6:**
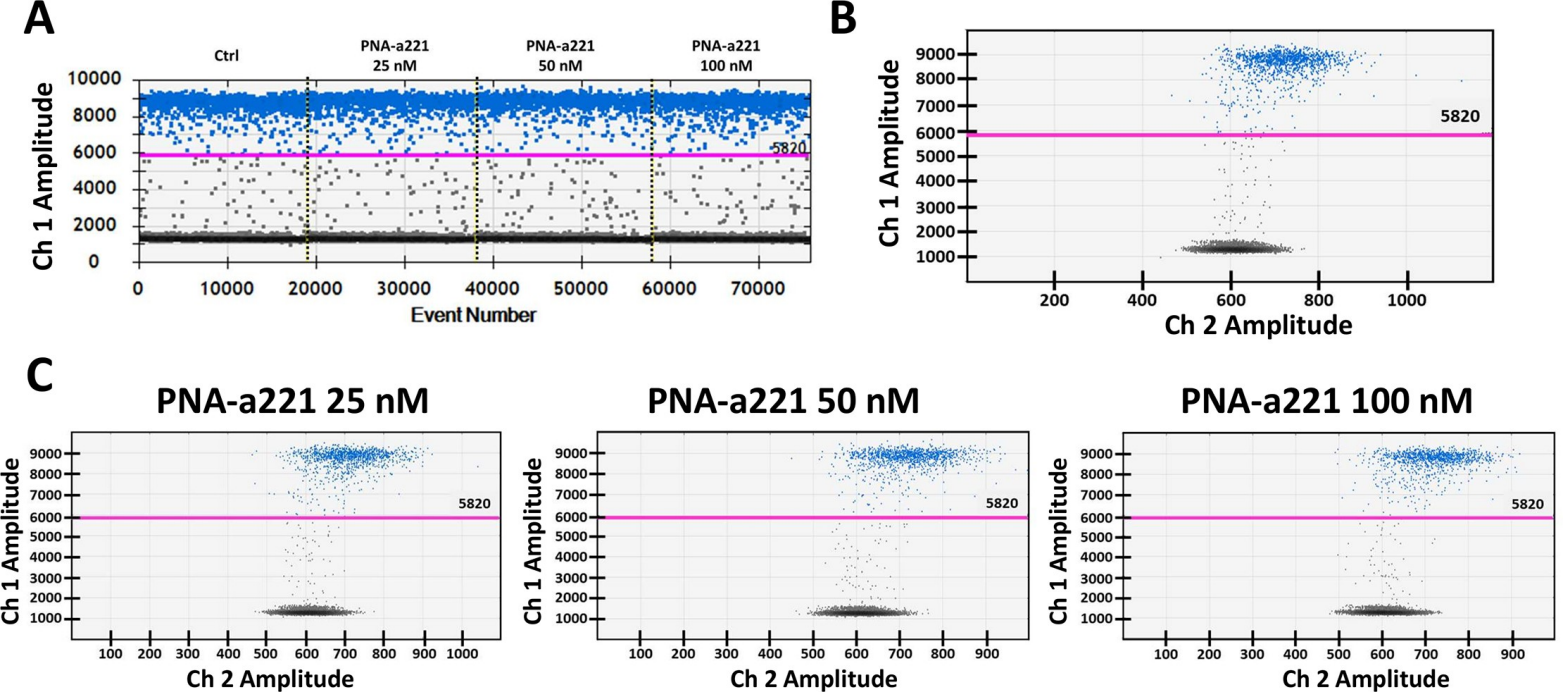
Effects of R8-PNA-a221 on miR-222-3p sequence detection by RT-ddPCR. A. 1D RT-ddPCR plot obtained after the addition of incremental concentration of R8-PNA-a221, miR-222-3p is amplified. B. miR-222-3p content in 1:50 diluted cDNA obtained from U251 cells: 2D plots. C. miR-222-3p content detected after the addition of incremental R8-PNA-a221concentrations.

Some of the experiments reported in Fig 2 were repeated using droplet digital PCR (ddPCR), another RT-PCR system routinely used for miRNA quantification [69]. Thanks to sample partition ddPCR allows the absolute miRNA sequences quantification with more precision compare to traditional RT-qPCR methods. Considering the dramatic reduction of miR-221-3p amplification detected by RT-qPCR when 200 nM of R8-PNA-a221 are employed, only three PNA concentrations were considered: 25, 50 and 100 nM. Obtained key results are similar to those obtained by RT-qPCR, with minor differences easily explained by the differences within miRNA quantification methods. In fact, while in RT-qPCR 25 nM of R8-PNA-a221 is sufficient to inhibit of 75% the miR-221-3p sequence amplification, when the same PNA concentration is employed in RT-ddPCR very limited effects (reduction of 17%) were detected, while more significative effects were recorded with 50 nM of PNA (35%) and 100 nM (100%) (Fig 5). Obtained data are quite expected considering sample and PNA partitioning in thousands of droplets. According to those obtained by RT-qPCR no effects of R8-PNA-a221 were detected on others miRNA sequences (i.e miR-222-3p) and no activity was founded when R8-PNA-a221-MUT was employed, even at highest concentration (100 nM) (Fig 6).

## Discussion

Simple experiments answering to key issues in applied pharmacology could be of great interest in the teaching, with particular focus to the possibility to set-up practical exercises in laboratory practice delivered to student in the field of biotechnology, pharmaceutics, applied biology.

One of the emerging pharmaceutical approaches is the so called miRNA therapy, based on antimiRNA strategy or on miRNA replacement, depending on the role covered by the target miRNA [11–14]. Alteration of microRNA expression has been demonstrated to be associated with different human pathologies [69–75], as well as guided alterations of specific miRNAs have been suggested as novel approaches to develop innovative therapeutic protocols [39,41,42,76–78]. Several reports conclusively demonstrated that microRNA are deeply involved in tumor onset and progression, behaving as tumor promoting miRNAs (oncomiRNA and metastamiRNAs) as well as tumor suppressor miRNAs [79–83]. In general, a miRNA able to promote cancer targets mRNA coding for tumor-suppression proteins, while microRNAs exhibiting tumor-suppression properties usually target mRNAs coding oncoproteins [14,84,85].

As far as the antimiRNA therapy, among the most interesting biomolecules to be analyzed are peptide-nucleic acids (PNAs). These are in fact reagents of great impact in antisense therapy, and have been proposed in a large spectrum of applications.

For instance, PNAs have been recently proposed as antisense molecules targeting mRNAs, as molecules able to target gene promoters through the formation of triple-helix structures, artificial promoters, or decoy molecules able to target transcription factors [17,47,85–89].

In this manuscript we present simple experiments that can be the basis for laboratory practical teaching with the aim of determining: (a) the possible PNA-mediated arrest in RT-qPCR, to be eventually used to demonstrate PNA targeting of selected miRNAs; (b) the possible lack of activity on mutated PNA sequences; (c) the effects (if any) on the amplification of other unrelated miRNA sequences.

The results which can be obtained during this laboratory teaching activity support the following conclusions: PNA-mediated arrest in RT-qPCR can be analyzed in a easy way; mutated PNA sequences are completely inactive; the effects of the employed PNAs are specific and no inhibitory effect occurs on other unrelated miRNA sequences.

This activity is simple (cell culture, RNA extraction, RT-qPCR are all well-established technologies), fast (starting from isolated and characterized RNA, few hours are just necessary), highly reproducible (therefore easily employed by even untrained students).

On the other hand, these laboratory lessons require some facilities, the most critical being the availability of instruments for PCR. While, this might be a problem in the case these instruments are not available, we would like to underline that determination of the presence or of a lack of amplified product can be also obtained using standard analytical approaches based on agarose gel electrophoresis.

## Supporting information

S1 File These Supplementary materials include some Figures that can be used for explaining the impact of PNAs in experimental therapeutic protocols (Figures A-C), the effects of PNAs against microRNAs miR-221-3p (Figure D) and miR-145-5p (Figure E). In addition, in the Supplementary Figure F the analysis of integrity of the RNA preparation is shown (see the protocols for the laboratory practice depicted in Figs 1 and 3 of the main text). Finally, Figures G and H report the inhibitory effects of the PNAs against miR-221-3p (PNA-a221 and PNA-a221-MUT, Figure G) and of the PNAs against miR-145-5p (PNA-a221 and PNA-a221-MUT, Figure H) on RT-qPCR amplification of the target miR-221-3p and miR-145-5p, and of the control miRNA sequences (miR-222-3p, let-7c-5p and miR-210-3p for PNA-a221; let-7c-5p and miR-155-5p for PNA-a145).
(DOCX)

## Abbreviations

PNA: peptide nucleic acid
miRNA: microRNA
RT-PCR: reverse transcription polymerase-chain reaction
