# Supplementary material for: Demonstrating specificity of bioactive peptide nucleic acids (PNAs) targeting microRNAs for practical laboratory classes of applied biochemistry and pharmacology
Source: PLoS One. 2019 Sep 11;14(9):e0221923. doi: 10.1371/journal.pone.0221923 (PMC6738603; doi:10.1371/journal.pone.0221923)
Supplement: S1 File — These Supplementary materials include some Figures that can be used for explaining the impact of PNAs in experimental therapeutic protocols (Figures A-C), the effects of PNAs against microRNAs miR-221-3p (Figure D) and miR-145-5p (Figure E). In addition, in the Supplementary Figure F the analysis of integrity of the RNA preparation is shown (see the protocols for the laboratory practice depicted in Figs 1 and 3 of the main text). Finally, Figures G and H report the inhibitory effects of the PNAs against miR-221-3p (PNA-a221 and PNA-a221-MUT, Figure G) and of the PNAs against miR-145-5p (PNA-a221 and PNA-a221-MUT, Figure H) on RT-qPCR amplification of the target miR-221-3p and miR-145-5p, and of the control miRNA sequences (miR-222-3p, let-7c-5p and miR-210-3p for PNA-a221; let-7c-5p and miR-155-5p for PNA-a145). (DOCX) [file pone.0221923.s001.docx]

Demonstrating specificity of bioactive peptide nucleic acids (PNAs) targeting microRNAs for practical laboratory classes of applied biochemistry and pharmacology

Jessica Gasparello^1^, Chiara Papi^1^, Matteo Zurlo^1^,

Roberto Corradini^2^, Roberto Gambari^1,3,*^ and Alessia Finotti^1^

^1^Department of Life Sciences and Biotechnology, University of Ferrara, Ferrara, Italy;

^2^Department of Chemistry, Life Sciences and Environmental Sustainability, University of Parma, Parma, Italy;

^3^Interuniversity Consortium for Biotechnology (CIB), Trieste, Italy

**Corresponding author*

E-mail: [gam@unife.it](mailto:gam@unife.it)

*Abbreviations:* PNA, peptide nucleic acid; miRNA, microRNA; RT-PCR, reverse transcription polymerase-chain reaction.

*Keywords:* Peptide nucleic acids, glioma, microRNAs, miRNA targeting, laboratory teaching.

**SUPPLEMENTARY MATERIAL**

**Introduction**

These Supplementary materials include some Figures that can be used for explaining the impact of PNAs in experimental therapeutic protocols (Figures A-C), the effects of PNAs against microRNAs miR-221-3p (Figure D) and miR-145-5p (Figure E). In addition, in the Supplementary Figure F the analysis of integrity of the RNA preparation is shown (see the protocols for the laboratory practice depicted in Figures 1 and 3 of the main text). Finally, Figures G and H report the inhibitory effects of the PNAs against miR-221-3p (PNA-a221 and PNA-a221-MUT, Figure G) and of the PNAs against miR-145-5p (PNA-a221 and PNA-a221-MUT, Figure H) on RT-qPCR amplification of the target miR-221-3p and miR-145-5p, and of the control miRNA sequences (miR-222-3p, let-7c-5p and miR-210-3p for PNA-a221; let-7c-5p and miR-155-5p for PNA-a145).


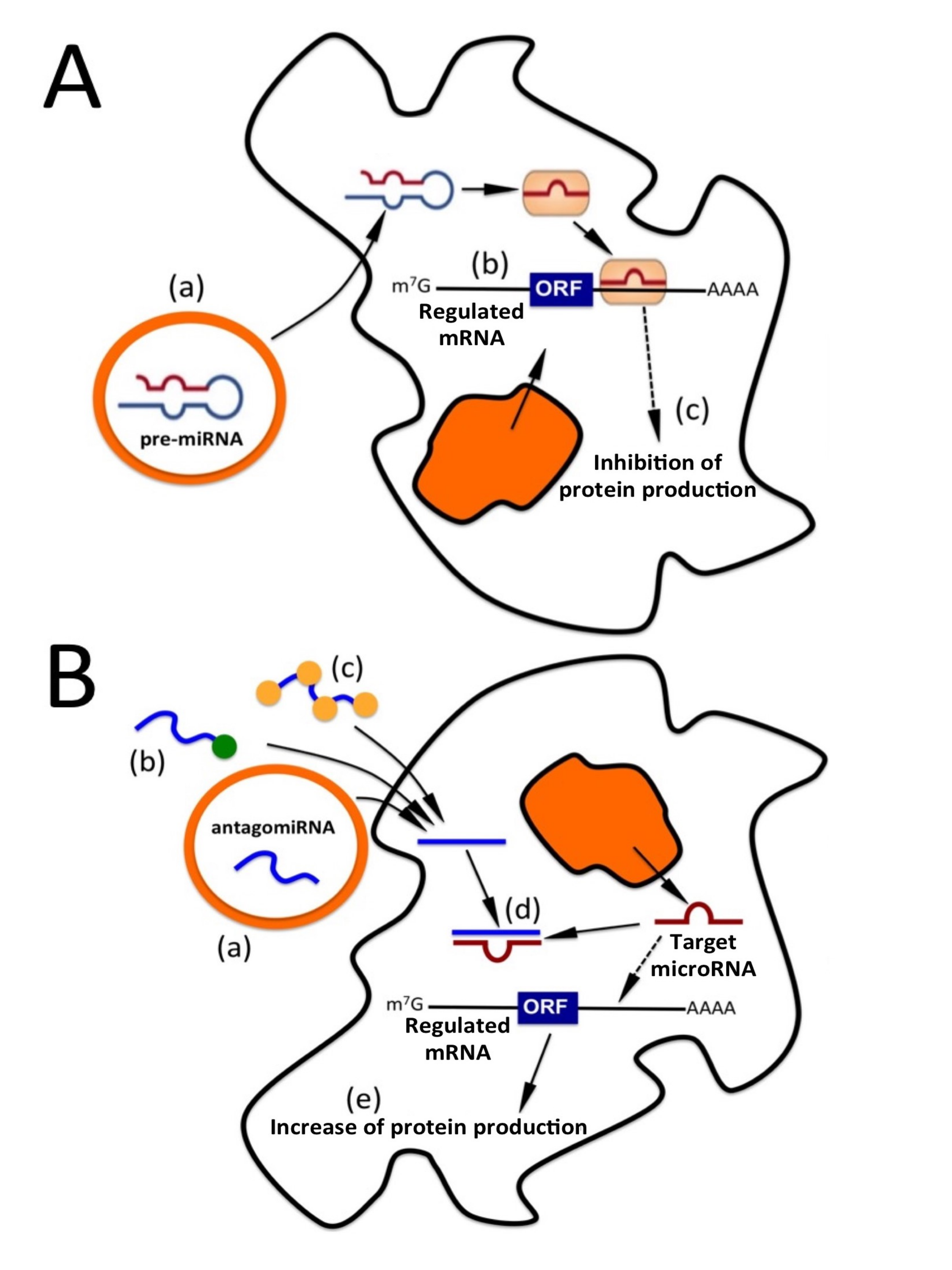


**Figure A. Scheme summarizing the miRNA replacement and anti-miRNA approaches to modify miRNA-regulated gene expression.** In panel A the miRNA replacement molecule is transfected to target cells (a) where interact with the mRNA to be modulated (b) causing inhibition of protein production (c, dotted arrow). In panel B miRNA inhibitors (a-c) are transfected to target cells (d) where they interact with the microRNA target preventing its binding to the specific 3’UTR sequence (dotted arrow) of the regulated mRNA (d). This causes up-regulation of this mRNA with increased protein production (e). Three examples of antagomiRNA molecules are shown: microparticle delivered antagomiRNAs (a), peptide-delivered molecules (b, peptide in green), or chemically-modified molecules (chemical modifications in yellows) to increase biological functions (for instance resistance to enzymatic degradation or delivery efficiency to target cells). Adapted from Gambari et al. [1].

**
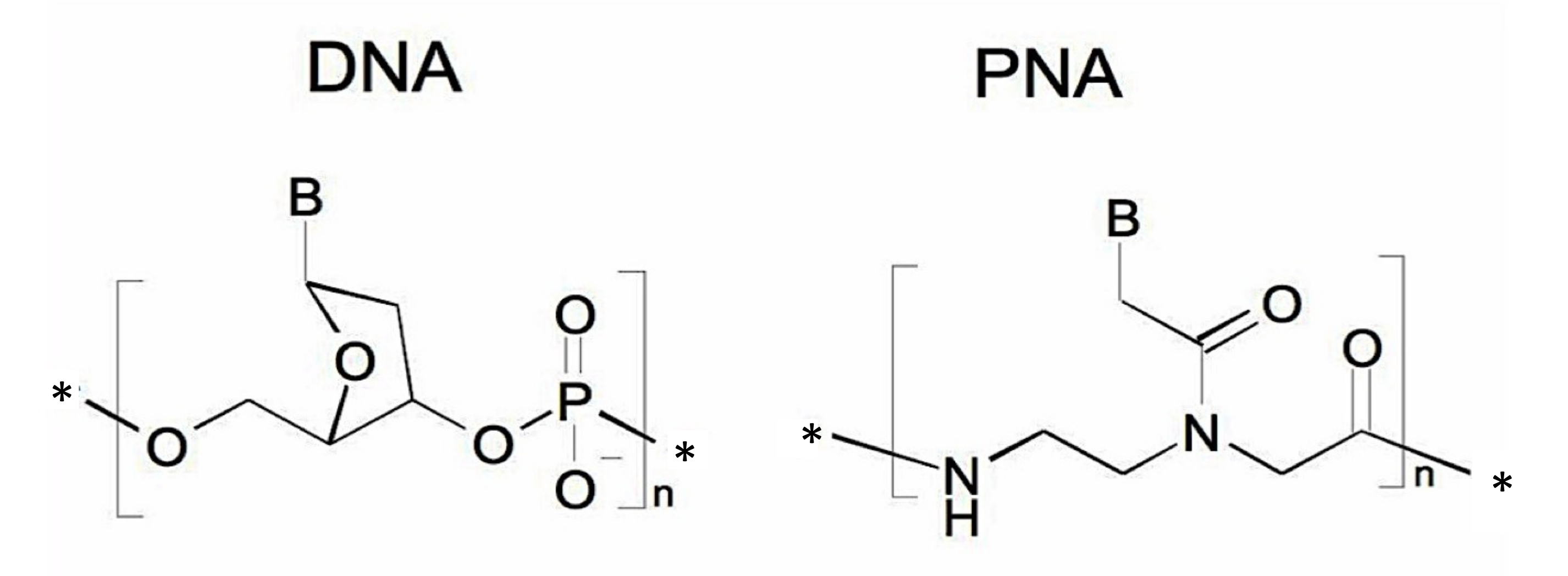
**

**Figure B. The general structure of PNA monomers.** Scheme outlining the differences between DNA (left) and PNA (right) monomers.

**
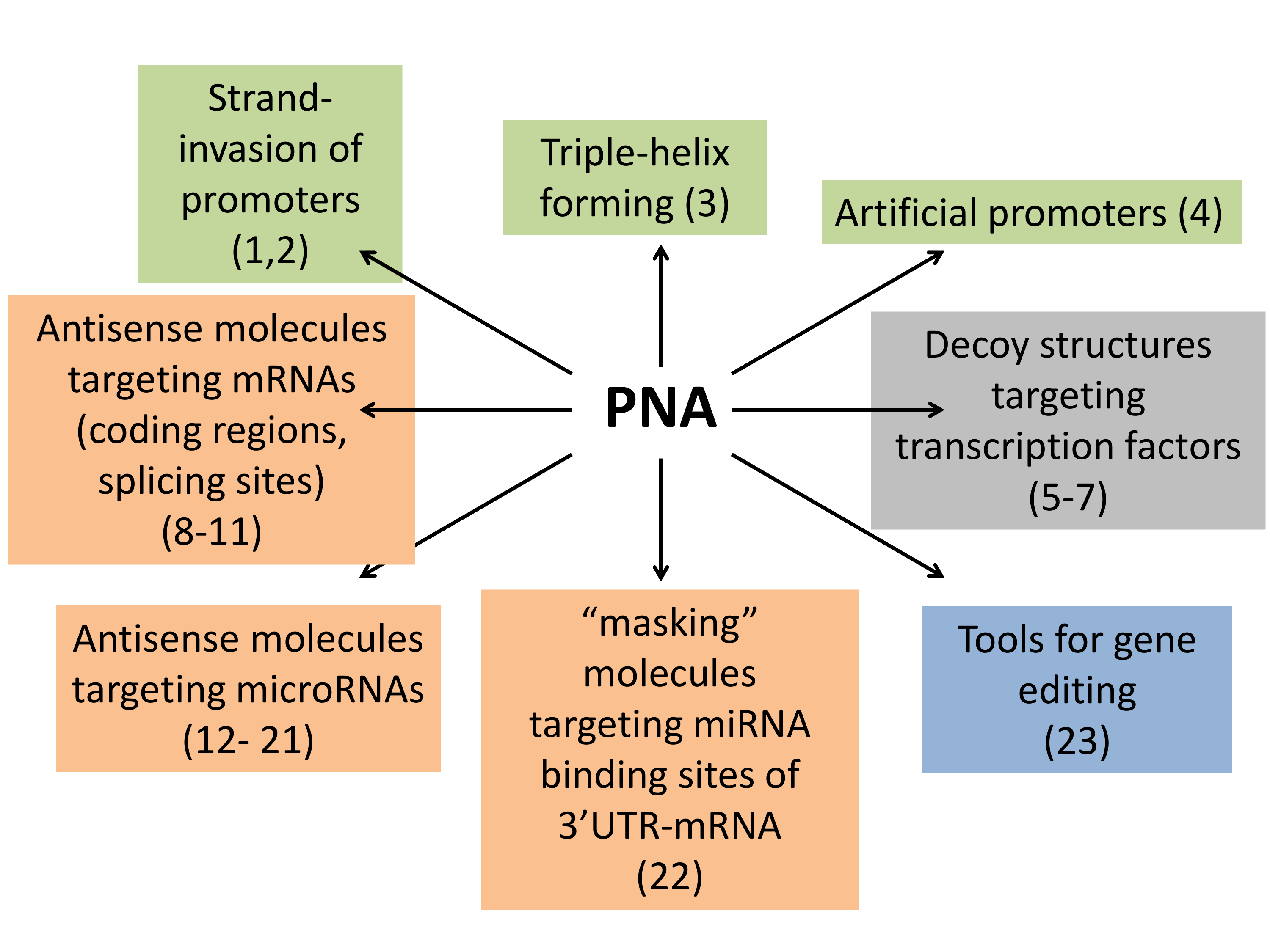
**

**Figure C. Effects of biologically-active peptide nucleic acids.** Representative manuscripts are shown (1-23).

**
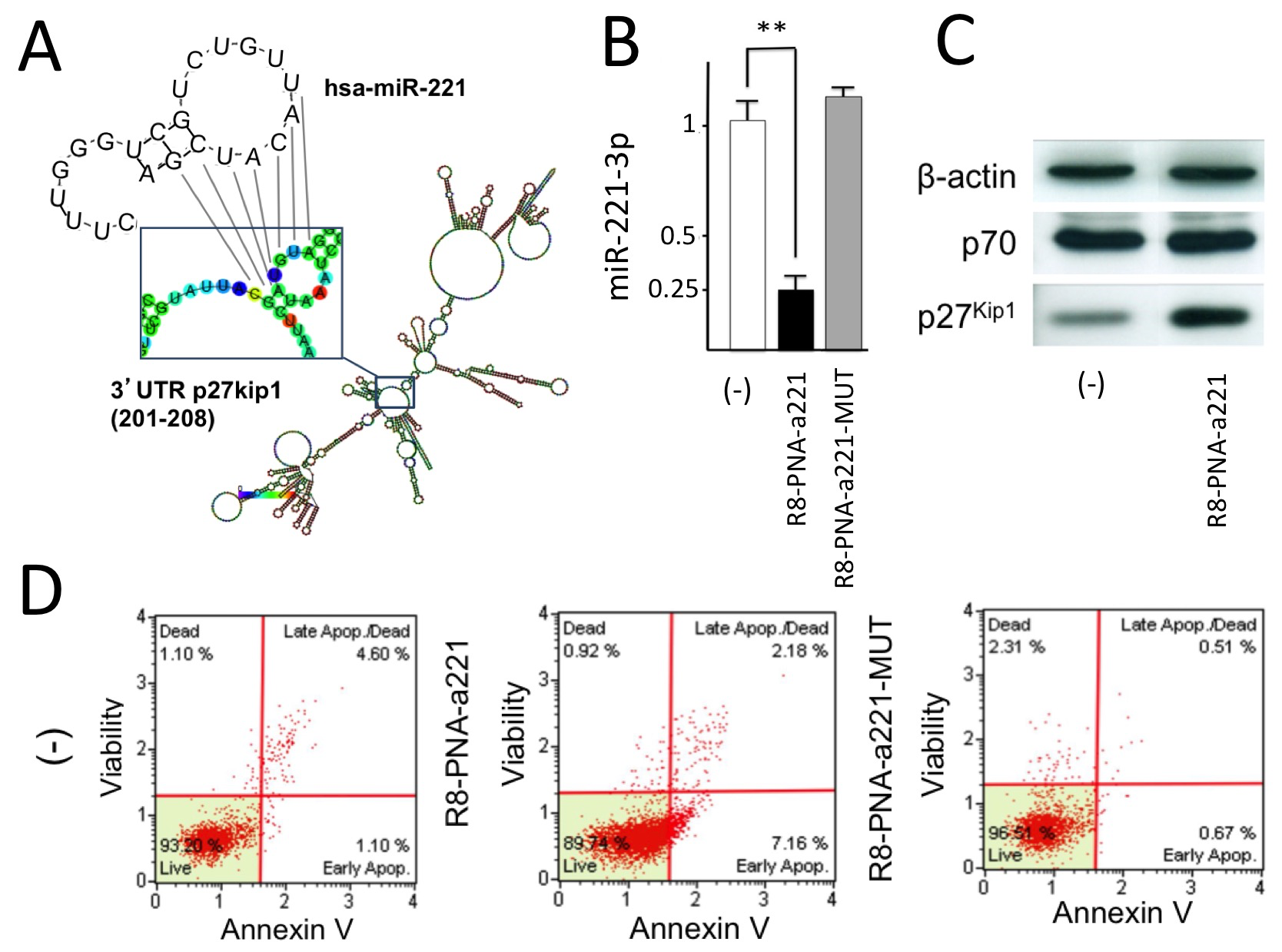
**

**Figure D. Biological effects of a PNA targeting miR-221-3p.** A. Structures of miR-221 and the 3’-UTR sequences of human p27^Kip1^ mRNA, showing the interactions between miR-221 and p27^Kip1^ mRNA. B. Effects of treatment of U251 with 2 µM R8-PNA-a221 and 2 µM R8-PNA-a221-MUT as indicated. The effects on hybridization to probes recognizing miR-221-3p are shown. C. Expression of p27^kip1^ in U-251 glioma cells treated for 96 h with R8-PNA-a221: Western blotting analysis performed using antibodies against p27^kip1^ and against β-actin, used as reference protein. D. Levels of apoptosis after treatment of U251 with 2 µM R8-PNA-a221 and 2 µM R8-PNA-a221-MUT as indicated. Modified from Brognara et al., 2014 [17].

**
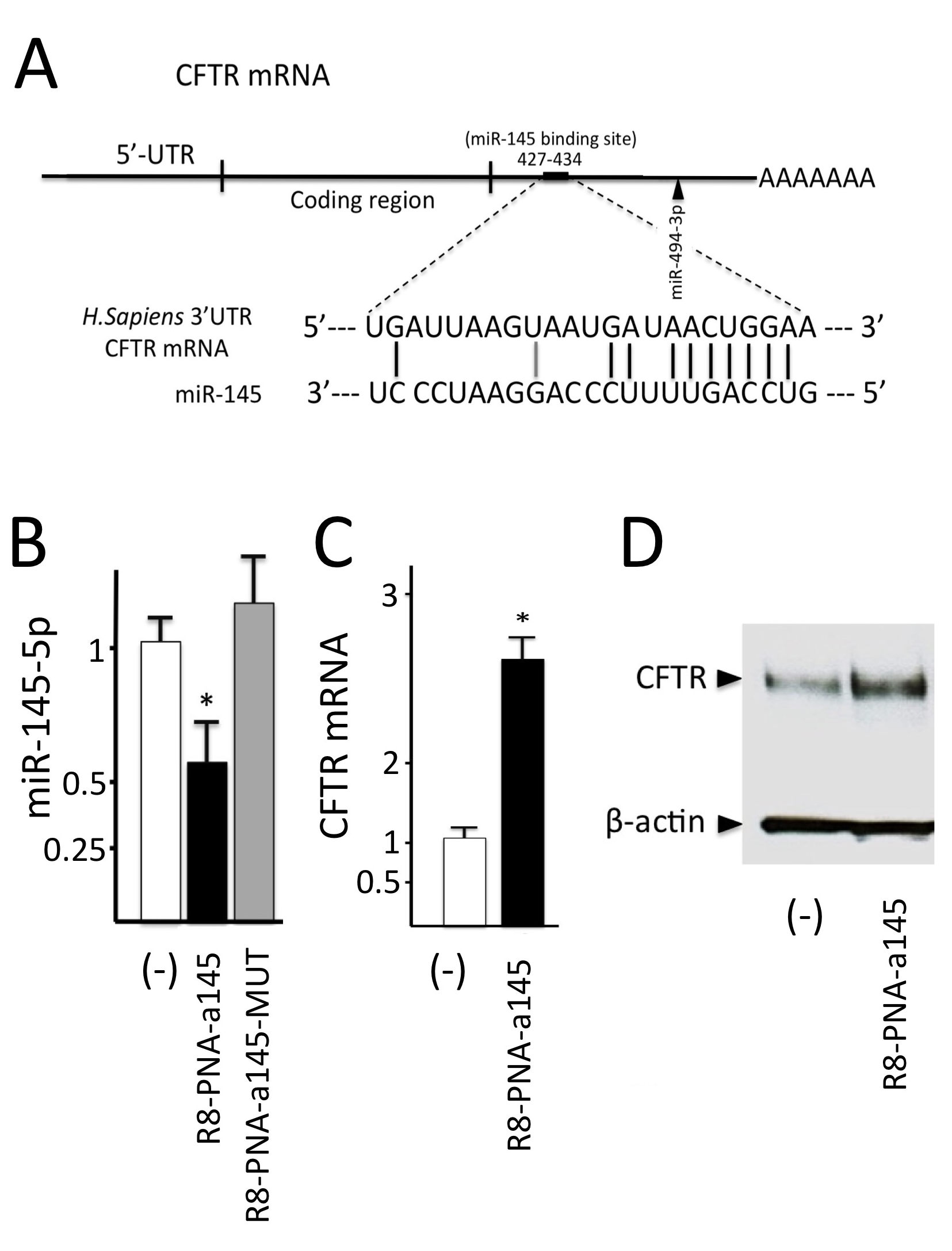
**

**Figure E. Biological effects of a PNA targeting miR-145-5p.** A. Location of the miR-145-5p binding sites within the CFTR 3’-UTR region. B. Inhibition of miR-145-5p hybridization signals in Calu-3 cells treated for 72 hours with R8-PNA-a145 or mutated R8-PNA-a145-MUT (2 μM, as indicated). Results shown represent the average ± S.D. obtained in at least three independent experiments. * = p<0.05. C,D. Upregulation of CFTR mRNA (RT-qPCR, C; N = 3; * = p<0.05) and CFTR protein (Western blotting, D) in Calu-3 cells cultured for 72 hours in the absence or in the presence of R8-PNA-a145-5p. CFTR and β-actin is indicated in panel D with arrowheads. Modified from Fabbri et al. [20].


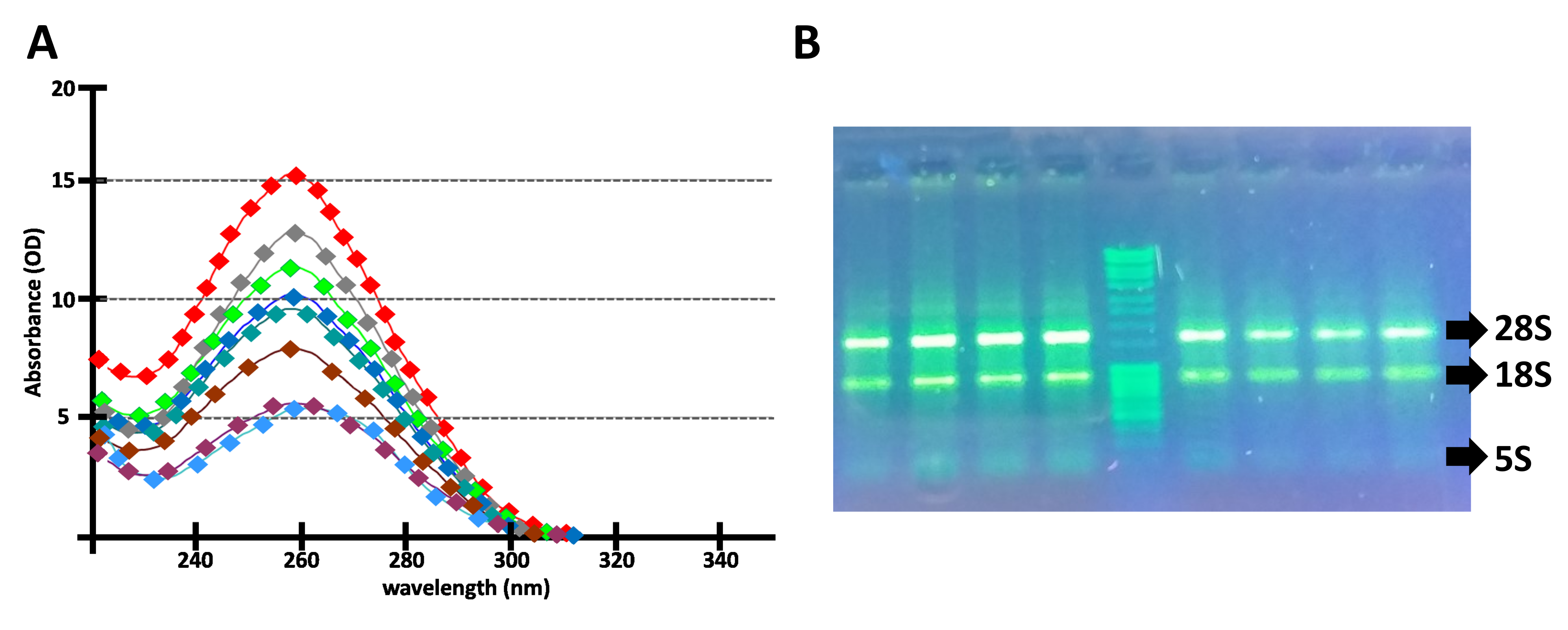


**Figure F. Analysis of the quality of the extracted RNA.** A. Spectrophotometric analysis. B. Integrity of the RNA preparations assayed by agarose gel-electrophoresis.

**
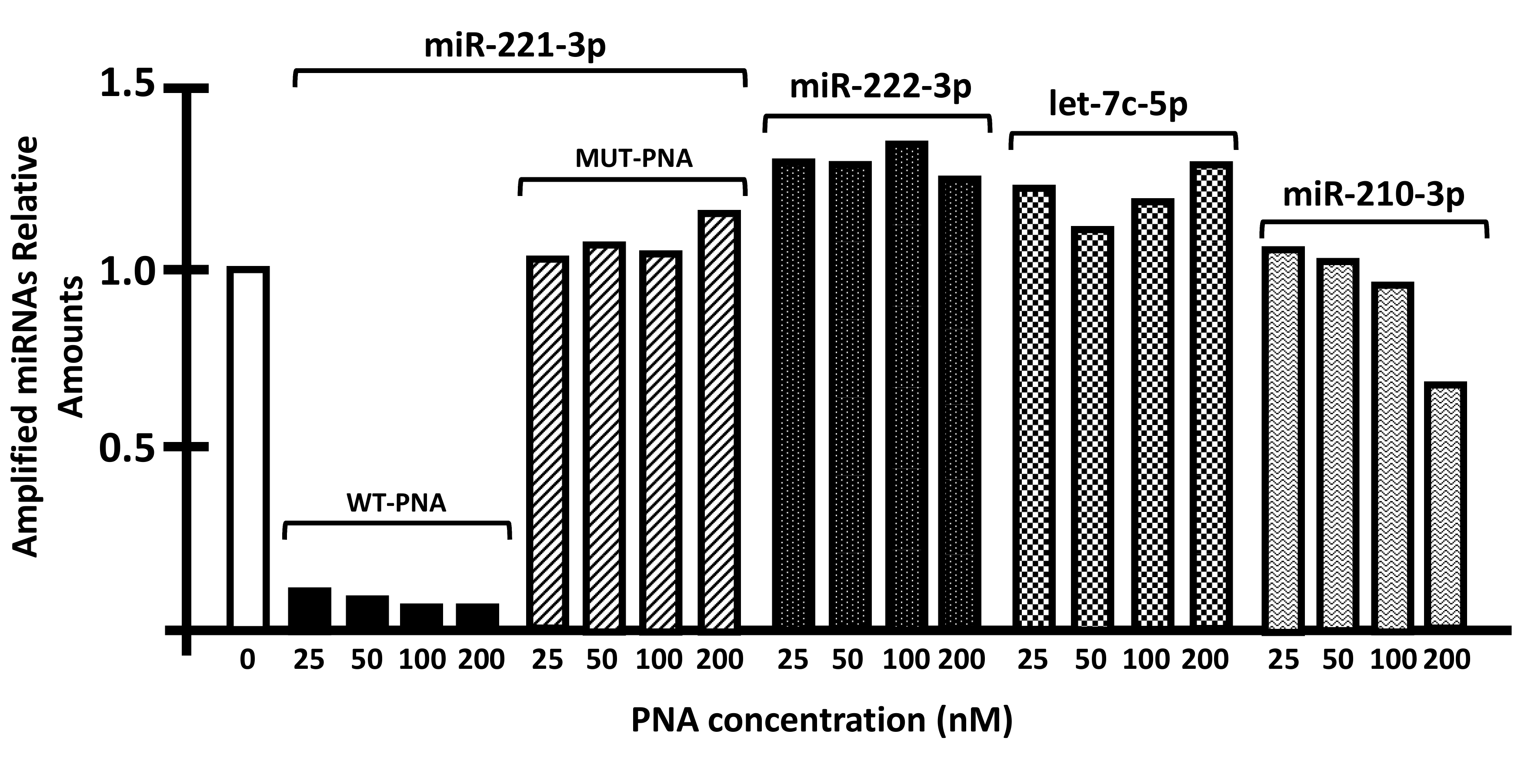
**

**Figure G. Effects of the PNA-a221 on the RT-PCR amplification of miRNA sequences** Effects of the PNAs against miR-221-3p (PNA-a221 and PNA-a221-MUT) used at the indicated concentration) on RT-qPCR amplification of miR-221-3p, miR-222-3p, let-7c-5p and miR-210-3p sequences.

**
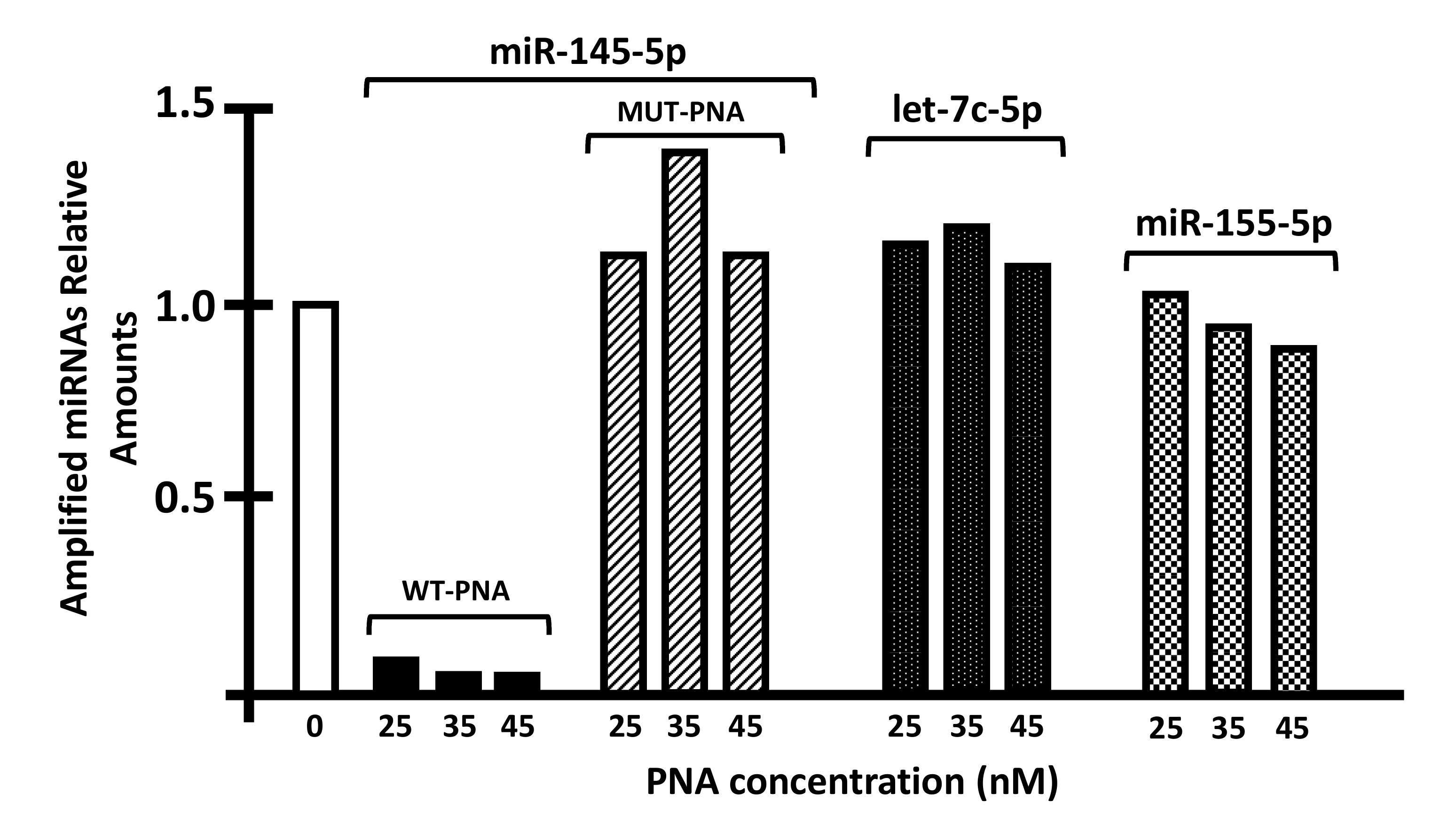
**

**Figure H. Effects of the PNA-a145 on the RT-PCR amplification of miRNA sequences.** Effects of the PNA against miR-145-5p (PNA-a145 and PNA-a145-MUT) used at the indicated concentration) on RT-qPCR amplification of miR-145-5p, let-7c-5p and miR-155-5p sequences.

**References**

1. Gambari R, Brognara E, Spandidos DA, Fabbri E. Targeting oncomiRNAs and mimicking tumor suppressor miRNAs: Νew trends in the development of miRNA therapeutic strategies in oncology (Review). Int J Oncol. 2016;49:5-32.

2. Li W, Shi H, Dong B, Nie K, Liu Z, He N. Recognition Mechanisms and Applications of Peptide Nucleic Acids Targeting Double-stranded DNA. Curr Med Chem. 2016;23: 4681-4705.

3. Endoh T, Hnedzko D, Rozners E, Sugimoto N. Nucleobase-Modified PNA Suppresses Translation by Forming a Triple Helix with a Hairpin Structure in mRNA In Vitro and in Cells. Angew Chem Int Ed Engl. 2016;55: 899-903.

4. Møllegaard NE, Buchardt O, Egholm M, Nielsen PE. Peptide nucleic acid.DNA strand displacement loops as artificial transcription promoters. Proc Natl Acad Sci U S A. 1994;91: 3892-3895.

5. Gambari R, Borgatti M, Bezzerri V, Nicolis E, Lampronti I, Dechecchi MC, et al. Decoy oligodeoxyribonucleotides and peptide nucleic acids-DNA chimeras targeting nuclear factor kappa-B: inhibition of IL-8 gene expression in cystic fibrosis cells infected with Pseudomonas aeruginosa. Biochem Pharmacol. 2010;80: 1887-1894.

6. Borgatti M, Lampronti I, Romanelli A, Pedone C, Saviano M, Bianchi N, etaMischiati, C. & Gambari, R. Transcription factor decoy molecules based on a peptide nucleic acid (PNA)-DNA chimera mimicking Sp1 binding sites. J Biol Chem. 2003;278: 7500-7509.

7. Romanelli A, Pedone C, Saviano M, Bianchi N, Borgatti M, Mischiati C et al. Molecular interactions with nuclear factor kappaB (NF-kappaB) transcription factors of a PNA-DNA chimera mimicking NF-kappaB binding sites. Eur J Biochem. 2001;268: 6066-6075.

8. Turner JJ, Fabani M, Arzumanov AA, Ivanova G, Gait MJ. Targeting the HIV-1 RNA leader sequence with synthetic oligonucleotides and siRNA: chemistry and cell delivery. Biochim Biophys Acta. 2005;1758: 290-300.

9. Montagner G, Bezzerri V, Cabrini G, Fabbri E, Borgatti M, Lampronti I, et al. An antisense peptide nucleic acid against Pseudomonas aeruginosa inhibiting bacterial-induced inflammatory responses in the cystic fibrosis IB3-1 cellular model system. Int J Biol Macromol. 2017;99: 492-498.

10. Ghosal A, Nielsen PE. Potent antibacterial antisense peptide-peptide nucleic acid conjugates against Pseudomonas aeruginosa. Nucleic Acid Ther. 2012;22: 323-334.

11. Abes S, Turner JJ, Ivanova GD, Owen D, Williams D, Arzumanov A, et al. Efficient splicing correction by PNA conjugation to an R6-Penetratin delivery peptide. Nucleic Acids Res. 2007;35: 4495-502.

12. Gambari R, Fabbri E, Borgatti M, Lampronti I, Finotti A, Brognara E, et al. Targeting microRNAs involved in human diseases: a novel approach for modification of gene expression and drug development. Biochem Pharmacol. 2011;82: 1416-1429.

13. Fabbri E, Brognara E, Borgatti M, Lampronti I, Finotti A, Bianchi N, et al. miRNA therapeutics: delivery and biological activity of peptide nucleic acids targeting miRNAs. Epigenomics. 2011;3: 733-745.

14. Fabbri E, Manicardi A, Tedeschi T, Sforza S, Bianchi N, Brognara E, et al. Modulation of the biological activity of microRNA-210 with peptide nucleic acids (PNAs). ChemMedChem. 2011;6: 2192-202.

15. Brognara E, Fabbri E, Bianchi N, Finotti A, Corradini R, Gambari R. Molecular methods for validation of the biological activity of peptide nucleic acids targeting microRNAs. Methods Mol Biol. 2014;1095: 165-176.

16. Brognara E, Fabbri E, Aimi F, Manicardi A, Bianchi N, Finotti A, et al. Peptide nucleic acids targeting miR-221 modulate p27Kip1 expression in breast cancer MDA-MB-231 cells. Int J Oncol. 2012;41: 2119-2127.

17. Brognara E, Fabbri E, Bazzoli E, Montagner G, Ghimenton C, Eccher A, et al. Uptake by human glioma cell lines and biological effects of a peptide-nucleic acids targeting miR-221. J Neurooncol. 2014;118: 19-28.

18. Brognara E, Fabbri E, Montagner G, Gasparello J, Manicardi A, Corradini R, et al. High levels of apoptosis are induced in human glioma cell lines by co-administration of peptide nucleic acids targeting miR-221 and miR-222. Int J Oncol. 2016;48: 1029-1038.

19. Bertucci A, Prasetyanto EA, Septiadi D, Manicardi A, Brognara E, Gambari R, et al. Combined Delivery of Temozolomide and Anti-miR221 PNA Using Mesoporous Silica Nanoparticles Induces Apoptosis in Resistant Glioma Cells. Small. 2015;11: 5687-5695.

20. Fabbri E, Tamanini A, Jakova T, Gasparello J, Manicardi A, Corradini R, et al. A Peptide Nucleic Acid against MicroRNA miR-145-5p Enhances the Expression of the Cystic Fibrosis Transmembrane Conductance Regulator (CFTR) in Calu-3 Cells. Molecules. 2017;23: E71.

21. Finotti A, Gasparello J, Fabbri E, Tamanini A, Corradini R, Dechecchi MC, et al. Enhancing the Expression of CFTR Using Antisense Molecules Against MicroRNA miR-145-5p. Am J Respir Crit Care Med. 2019; In press.

22. Zarrilli F, Amato F, Morgillo CM, Pinto B, Santarpia G, Borbone N, et al. Peptide Nucleic Acids as miRNA Target Protectors for the Treatment of Cystic Fibrosis. Molecules. 2017;22: E1144.

23. Ricciardi AS, Quijano E, Putman R, Saltzman WM, Glazer PM. Peptide Nucleic Acids as a Tool for Site-Specific Gene Editing. Molecules. 2018;23: E632.
